# Inhibitory Effect of Guava Leaf Polyphenols on Advanced Glycation End Products of Frozen Chicken Meatballs (−18 °C) and Its Mechanism Analysis

**DOI:** 10.3390/foods11162509

**Published:** 2022-08-19

**Authors:** Mengna Zhao, Ying Li, Xue Bai, Jia Feng, Xiufang Xia, Fangfei Li

**Affiliations:** 1 College of Food Science, Northeast Agricultural University, Harbin 150030, China; 2 College of Forestry, Northeast Forestry University, Harbin 150040, China

**Keywords:** guava leaf polyphenols, advanced glycation end products, lipid oxidation, protein oxidation, Maillard reaction

## Abstract

The inhibitory effect of guava leaf polyphenols (GLP) on advanced glycation end products (AGEs) of frozen chicken meatballs (−18 °C) and its possible inhibitory mechanism was investigated. Compared with control samples after freezing for 6 months, acidic value (AV), lipid peroxides, thiobarbituric acid reactive substance (TBARS), A_294_, A_420_, glyoxal (GO), N^ε^-carboxymethyl-lysine (CML), pentosidine, and fluorescent AGEs of chicken meatballs with GLP decreased by 11.1%, 22.3%, 19.5%, 4.30%, 8.66%, 8.27%, 4.80%, 20.5%, and 7.68%, respectively; while free sulfhydryl groups the content increased by 4.90%. Meanwhile, there was no significant difference between meatballs with GLP and TP in AV, A_294_, GO, and CML (*p* > 0.05). Correlation analysis indicated that GO, CML, pentosidine, and fluorescent AGEs positively correlated with AV, TBARS, A_294_, and A_420_, while GO, CML, pentosidine, and fluorescent AGEs negatively correlated with free sulfhydryl groups. These results manifested GLP could inhibit AGEs formation by inhibiting lipid oxidation, protein oxidation, and Maillard reaction. The possible inhibitory mechanism of GLP on the AGEs included scavenging free radicals, capturing dicarbonyl compounds, forming polyphenol–protein compounds, and reducing the formation of glucose. Therefore, the work demonstrated that the addition of plant polyphenols may be a promising method to inhibit AGEs formation in food.

## 1. Introduction

Chicken meatball, as a chicken processed meat product, is popular for its pleasant flavor, convenient processing, and low price, and is usually preserved at −18 °C to reduce the contamination of enzymes and microorganisms and prolong the shelf life [1,2,3]. However, low temperature does not completely inhibit enzyme activity [4]; at the same time, ice crystals give rise to physical damage of the muscle cells, which makes the pro-oxidation factors (free radicals) and enzymes (protease, and lipase) liberate [5,6], resulting in lipid and protein oxidation. Lipid oxidation generates dicarbonyl compounds that can participate in the Maillard reaction, causing the formation of advanced glycation end products (AGEs) [7]. It has been reported that the accumulation of AGEs in the body is linked to several diseases including kidney failure, diabetes, atherosclerosis, tumors, Alzheimer’s disease, and so on [8,9]. Therefore, it is necessary to inhibit the formation of AGEs in food.

At present, some inhibitors are used to inhibit the formation of AGEs, and they are divided into synthetic inhibitors and natural inhibitors according to the source. Clinically, synthetic aminoguanidine has been used as an inhibitor and has excellent inhibitory effects, but it is potentially toxic to humans [9]. In contrast, plant polyphenols are a kind of natural and safe inhibitor, and some polyphenols (such as tea polyphenol [8], black chokeberry polyphenol [9], bayberry tea polyphenol [10], and grape by-products extract [11]) have shown a potent inhibitory effect on the formation of AGEs due to their ability to scavenge free radicals.

Guava (*Psidium guajava* L.) is a member of the Myrtaceae family and is favored for its distinct flavor, nutritional components, and medicinal value [12]. The production of guava reached up to 55.85 million tons in 2019 worldwide [13]. In industry, the fruit has been used to produce juice, preserved fruit, jam [13], and so on because of its abundant nutritional and functional constituents (such as minerals, vitamin C, polyphenols, dietary fiber, carotenoids, etc.) [14,15], which lead to an amount of industry residues including peels, seeds, and leaves [16]. About 80 kg of residue is produced after one ton of guava is processed [13]. Among these residues, guava leaves are richer in polyphenol content than guava fruit, they are low-cost, and have a good ability to scavenge free radicals [17]. However, guava leaf polyphenols (GLP) have not been studied as inhibitors of the formation of AGEs in food.

Therefore, the aims of the present work were to (1) explore the effect of the GLP on the lipid and protein oxidation by acidic value (AV), lipid peroxides (POV), thiobarbituric acid-reactive substances (TBARS), and free sulfhydryl groups; (2) investigate the effect of the GLP on the degree of Maillard reaction by intermediate and advanced products; (3) evaluate the effect of the GLP on the AGEs by glyoxal (GO), N^ε^-carboxymethyl-lysine (CML), pentosidine, and fluorescent AGEs; (4) clarify the correlation of lipid oxidation, protein oxidation, Maillard reaction, and the formation of AGEs; and (5) analyze the possible inhibitory mechanism of the GLP on AGEs. Meanwhile, the hypothesis of this work is that the addition of GLP can inhibit AGE formation in frozen chicken meatballs (−18 °C) by inhibiting lipid oxidation, protein oxidation, and the Maillard reaction.

## 2. Materials and Methods

### 2.1. Materials

Fresh chicken breast samples were supplied by a local supermarket (Harbin, China). GLP (purity > 40%, 160.14 mg gallic acid equivalent/g) was purchased from Fufeng Snow Biotechnology Co. LTD (Baoji, China). Tea polyphenols (TP) (purity > 98%, 233.49 mg gallic acid equivalent/g) were obtained from Anhui Red Star Pharmaceutical Co. Ltd. (Xuancheng, China). All other reagents were supplied by Wantai Biomedicals Inc. (Harbin, China).

### 2.2. Preparation of Chicken Meatballs

The chicken meatballs were prepared according to Shahimi et al. with modifications [18]. Fresh chicken breasts and pork fat were ground and all ingredients were added according to Table 1. Among them, GLP and TP were added by dissolving them in water to prepare a GLP (450 mg/L) and TP (150 mg/L) solution. The GLP (450 mg/L) and TP (150 mg/L) mg/mL solution was stored at 4 °C before usage. Subsequently, the minced chicken was evenly mixed and molded into chicken meatballs about 30 mm in diameter and 20 g in weight, and then stored at −18 °C for 0, 1, 2, 3, and 6 months. The chicken meatballs without polyphenol, with GLP, and with TP were labeled as CON, GLP, and TP, respectively.

### 2.3. Oxidation Staility

#### 2.3.1. Acidic Value (AV)

The AV of the chicken meatballs during frozen storage was determined following Chinese Standard GB 5009.229-2016.

#### 2.3.2. Lipid Peroxides (POV)

The content of lipid peroxides in chicken meatballs was measured as described by Pan et al. [19]. The minced meat (2 g) was mixed with 20 mL chloroform: methanol solution (2:1, *V/V*) in a 50 mL centrifuge tube. The mixture was homogenized at 11,000 rpm for 30 s by using a M133/1281–0 high-shear mixer (Biospec Products, Bartlesville, OK, USA) and then 3 mL 0.5% NaCl solution was added to the mixture. The mixture was centrifuged at 5000 rpm for 10 min and, subsequently, the upper liquid phase was extracted using a 5 mL sterile syringe and injected into another 50 mL centrifuge tube. Then, 5 mL of chloroform: methanol solution (2:1, *V/V*) stored at 4 °C, 25 μL ammonium thiocyanate solution (30%, *W/V*), and 25 μL ferrous chloride solution were added into the centrifuge tube, and the solution mixed by vortexing for 3 s with each addition. The absorbance of the mixed solution was measured at 500 nm after reaction for 5 min at room temperature. The standard curve was prepared by using a reduced iron. The POV values were expressed as meq/kg of meat.

#### 2.3.3. Thiobarbituric Acid-Reactive Substances (TBARS)

The TBARS value was evaluated according to the method reported by Li et al. [20]. Minced meat of chicken meatballs (2 g) was mixed with 3 mL of 1% thiobarbituric acid (TBA) solution and 17 mL of 2.5% trichloroacetic acid-HCl (TCA-HCl) solution. Three drops of butylated hydroxytoluene (BHT) were added into the mixture, and the mixture was heated in boiling water (100 °C) for 30 min. After the mixture was cooled to room temperature, the same volume of chloroform was mixed by vortexing with the same volume of the mixture for 1 min, followed by centrifugation at 3000 rpm for 10 min. The absorbance of the supernatant was recorded at 532 nm. The TBARS value was expressed as mg of malonaldehyde/kg of chicken meatballs:

$$\mathrm{TBARS}\;\left(\mathrm{mg}/\mathrm{kg}\right)=\frac{A_{532}}{m}\times 9.48$$

where A_532_ is the absorbance at 532 nm; m is the weight of chicken meatballs sample (g); and “9.48” is a derived constant from the dilution factor and the molar extinction coefficient (152,000 M^−1^ cm^−1^) of the red TBA reaction product.

#### 2.3.4. Free Sulfhydryl Groups

According to the method described by Zhang et al. [21], myofibrillar protein was obtained. The free sulfhydryl groups were measured according to Xia et al. and Xia et al. with slight modifications [22,23]. Then, 1 mL myofibrillar protein solution (2 mg/mL) was added into 8 mL tris-glycine solution (pH 8) to prepare the mixture solution, and the mixture solution was homogenized and centrifuged at 10,000 rpm for 5 min. Centrifuge supernatant (4.5 mL) was reacted with 0.5 mL 10 mM Ellman reagent for 30 min. The absorbance was documented at 412 nm and 540 nm. The molar extinction coefficient was 13,600 M^−1^ cm^−1^. The results were recorded as nmol/mg protein.

### 2.4. Degree of Maillard Reaction

The degree of Maillard reaction was measured according to the method reported by Zhu et al. [24]. The absorbance was read at 295 nm and 420 nm to reflect intermediate and advanced products of the Maillard reaction, respectively.

### 2.5. Advanced Glycation end Products

#### 2.5.1. Glyoxal (GO)

The measure of GO was conducted according to the method of Zhu et al. [24] with slight modifications. Minced meat of chicken meatballs (0.5 g) was mixed with 0.5 mL of 15 g/L sodium acetate solution and 0.5 mL of 15 g/L hydroxylamine hydrochloride. Subsequently, the mixture was treated in a water bath at 50 °C for 20 min, and then cooled to room temperature. The cooled mixture was poured into a 25 mL volumetric flask and PBS (0.2 M, pH 8.0) was added to increase the volume to 25 mL. The absorbance was read at 233 nm.

#### 2.5.2. N^ε^-Carboxymethyl-Lysine (CML)

CML was determined by enzyme-linked immunosorbent assay, and specific operations were slightly modified according to the method described by Zhu et al. [25]. Standard holes, sample holes, zero holes, and blank holes were marked in a plate. Among them, no samples were added to blank holes, and 50 µL diluted standard solution was added to standard holes and zero holes, and 50 µL sample solution was added to sample holes. Subsequently, 50 µL biotin antigen working solution was added to each hole, and holes were covered with sealing plate films after gently shaking. Next, the plate was placed in an incubator at 37 °C for 30 min and the plate was cleaned five times using a detergent solution. Subsequently, 50 µL avidin-hrp was added to each hole and the above-mentioned steps were repeated. Then, 50 µL of color-developing agent A was first added to each hole and then 50 µL of color developing agent B was added to each hole. The plate was placed in an incubator at 37 °C for 10 min after gently shaking. At the end of the reaction, 50 µL stop buffer was added to stop the reaction, and the solution color changed from blue to yellow. The absorbance was recorded at 450 nm.

#### 2.5.3. Pentosidine

The pentosidine content was determined based on the method described by Joglekar et al. [26] with modifications. The fluorescence intensity was documented at the emission wavelengths from 360 to 500 nm by using a F-4500 fluorescence spectrophotometer (Hitachi, Tokyo, Japan).

#### 2.5.4. Fluorescent AGEs

The fluorescent AGE content was determined based on the steps described by Wu et al. [27] with minor modifications. The fluorescence intensity was documented at the emission wavelengths from 360 to 380 nm by using an F-4500 fluorescence spectrophotometer (Hitachi, Tokyo, Japan).

### 2.6. Statistical Analysis

The data were expressed as mean values ± standard error (SE), and an analysis of variance (ANOVA) with Tukey’s multiple comparisons was performed to analyze data under the significance level (*p* < 0.05) using a Statistix 8.0 software with the General Linear Models procedure (Analytical Software, St. Paul, MN, USA). Figures were plotted by using an OriginPro 2021 (OriginLab, Northampton, MA, USA). Data regarding the oxidation and Maillard reaction of chicken meatballs during the frozen storage were analyzed using a mixed model according to the analytical method of Biffin, Smith, Bush, Collins, and Hopkins [28], in combination with the research contents of our study. In this model, the fixed terms included polyphenols addition (GLP and TP) as well as frozen storage time (0, 1, 2, 3, 6 months), and each replication was included as a random term in this model.

## 3. Results and Discussion

### 3.1. Oxidation Stability

#### 3.1.1. Lipid Oxidation Stability

The effect of GLP on the lipid oxidation stability of chicken meatballs was evaluated by AV, POV, and TBARS. The AV, POV, and TBARS values can be used to evaluate the degree of lipid hydrolysis, fatty acids primary oxidation, and secondary oxidation, respectively. The higher the value, the greater the lipid hydrolysis and oxidation degree of the chicken meatballs.

**Figure 1:**
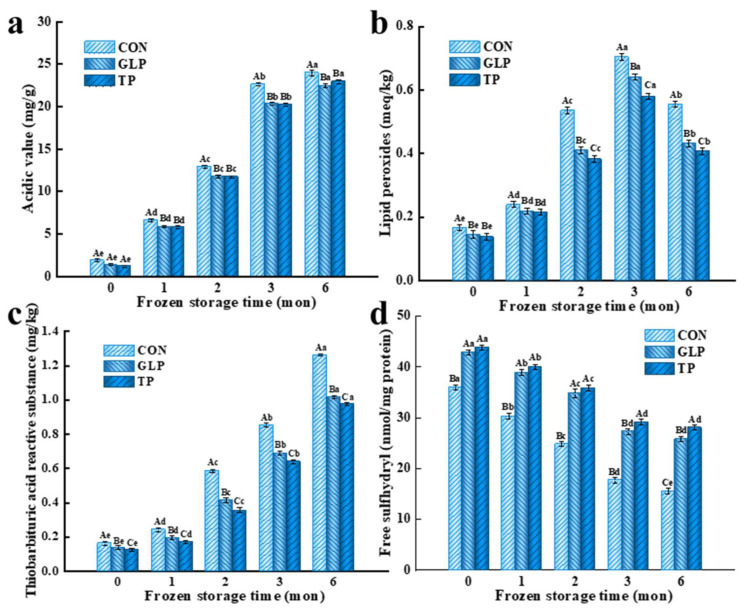
Effect of guava leaf polyphenols on the oxidation stability of frozen chicken meatballs. (**a**), Acidic value; (**b**), Lipid peroxides; (**c**), Thiobarbituric acid reactive substances; (**d**), Free sulfhydryl. GLP, guava leaf polyphenols; TP, tea polyphenol; CON, control. The means at the same frozen storage with different uppercase letters (A–C) differ significantly (*p* < 0.05); the means at the same polyphenol processing with different lowercase letters (a–e) differ significantly (*p* < 0.05). The results are mean ± SE (*n* = 3).

As shown in Figure 1a, with the extension of frozen storage time, AV values of all chicken meatballs significantly increased (*p* < 0.05). Compared with the control samples, AV values of chicken meatballs with GLP decreased by 12.13%, 13.88%, 27.10%, and 11.08% at 1, 2, 3, and 6 months, respectively, and there was no significant difference compared to samples with TP (*p* > 0.05). As shown in Figure 1b, the POV values of all samples dramatically increased in the first three months, and significantly reduced after freezing for 6 months (*p* < 0.05). Compared with the control samples, POV values of the chicken meatballs with GLP decreased by 12.84%, 8.36%, 23.4%, 8.99%, and 22.3% at 0, 1, 2, 3, and 6 months, respectively. Compared to samples treated with TP, there was no obvious difference in the first month (*p* > 0.05). As shown in Figure 1c, with the extension of frozen storage time, TBARS values of all chicken meatballs significantly increased (*p* < 0.05). Compared with the control samples, TBARS values of chicken meatballs treated with GLP decreased by 14.21%, 19.44%, 29.42%, 19.33%, and 19.52% at 0, 1, 2, 3, and 6 months, respectively, while they significantly increased compared with samples with TP (*p* < 0.05).

The increase in AV values of chicken meatballs was attributed to the fact that ice crystals caused muscle cell rupture during frozen storage and then released a large number of pro-oxidant substances and enzyme, which would make lipid oxidize and hydrolyze into free fatty acids; therefore, the lipid hydrolysis degree of chicken meatballs increased [1]. The rise in POV values in the first three months was due to the free fatty acids generated by the breakdown of lipid oxidizing to produce hydrogen peroxide first, and then hydrogen peroxide was oxidized to POV, resulting in a large accumulation of POV at this stage. However, POV generated in the early stage of lipid oxidation was extremely unstable, and would be decomposed into small molecules, such as aldehydes, ketones, acids, and hydroxyl acids in the later stage of oxidation [29]. In other words, the synthesis rate of POV was higher than the decomposition rate in the first three months during frozen storage, while the decomposition rate of POV was higher than the synthesis rate after freezing for 6 months, causing the reduced POV values. The increase in the TBARS values of chicken meatballs was associated with the production of secondary oxidation products generated by the breakdown of POV [30]. The decrease in AV, POV, and TBARS of chicken meatballs with GLP compared with control samples was attributed to the quercetin, morin, quercetin-3-O-glucopyranoside, and other polyphenols present in the GLP. On the one hand, these polyphenols scavenged free radicals produced by lipid oxidation. On the other hand, they served as electron donors that reduced the oxidation intermediates in lipid peroxidation process to stable forms, thus terminating the oxidation chain reaction and inhibiting the primary and secondary oxidation of free fatty acid [31]. Similar phenomena were reported by Zwolan et al. [32], i.e., Nigella sativa L. seed extracts slowed the process of lipid oxidation for chicken meatballs during refrigerated storage.

#### 3.1.2. Protein Oxidation Stability

Protein oxidation stability can be reflected by free sulfhydryl group contents that are the most easily affected functional groups in chicken protein, and most of those (70%) are contained in MP [33]. The higher the free sulfhydryl groups content, the better the protein oxidation stability. As shown in Figure 1d, with the increase of frozen storage time, the free sulfhydryl groups content of all samples significantly decreased (*p* < 0.05), while the free sulfhydryl groups content of samples with GLP and TP increased by 4.90% and 6.95% compared with control samples after freezing for 6 months. During frozen storage, protein was oxidized, which destroyed sulfhydryl groups and made them convert to disulfide bonds, resulting in a decrease in sulfhydryl content [34,35]. After GLP and TP were incorporated, polyphenols with antioxidant activity reduced the production of reactive oxygen species and scavenged free radicals, thereby preventing the oxidation of protein sulfhydryl groups, resulting in the decrease of free sulfhydryl loss [36]. Moreover, polyphenols in GLP combined with protein to form polyphenol–protein compounds, which avoided protein being oxidized [11]. Huang et al. [37] also reported similar results, i.e., mulberry polyphenols decreased sulfhydryl content loss by scavenging free radicals; therefore, the protein oxidation stability was improved.

### 3.2. Degree of Maillard Reaction

#### 3.2.1. Intermediate Products of Maillard Reaction

**Figure 2:**
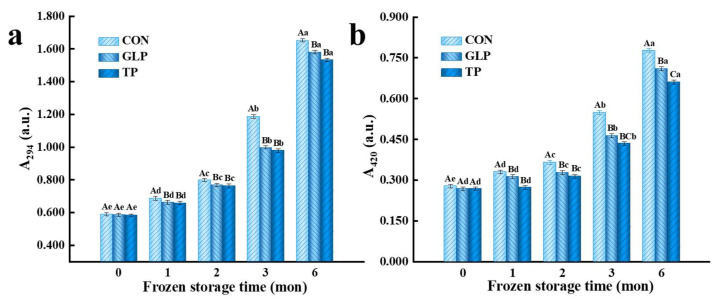
Effect of guava leaf polyphenols on the Maillard reaction of frozen chicken meatballs. (**a**), A_294_; (**b**), A_420_. GLP, guava leaf polyphenols; TP, tea polyphenol; CON, control. The means at the same frozen storage with different uppercase letters (A–C) differ significantly (*p* < 0.05); the means at the same polyphenol processing with different lowercase letters (a–e) differ significantly (*p* < 0.05). The results are mean ± SE (*n* = 3).

The Maillard reaction can produce small, colorless, intermediate products, including sugars, aldehydes, and ketones, which is reflected by the absorbance of samples at 294 nm (A_294_) [38]. The higher the A_294_, the higher the intermediate product’s content. As depicted in Figure 2a, the A_294_ of all meatballs significantly increased with the increase of frozen storage time; among them, the A_294_ of samples with GLP and TP significantly decreased compared with control samples after 1 month (*p* < 0.05). The increase in A_294_ was on account of the protein structure of chicken meatballs being unfolded, resulting from protein oxidation during frozen storage, and the inherent amino groups inside were exposed, and further reacted with carbonyl groups in residual sugars, causing the increased degree of the Maillard reaction [33]. The decrease in A_294_ was attributed to both GLP and TP inhibiting the protein oxidation and reducing the exposure amino groups, resulting in the decreased degree of Maillard reaction [36]. The results were similar to those of Zhu et al. [8], who revealed that catechins acting as a kind of phenolic substance had a superior inhibitory effect on early Maillard reactions.

#### 3.2.2. Advanced Products of the Maillard Reaction

At the advanced stage of the Maillard reaction, fragments of protein polypeptides and polysaccharides condense to form brown melanoidin, whose content can be evaluated by the absorbance of samples at 420 nm (A_420_) [38,39]. The higher the A_420_, the higher the melanoidin content, and the more thorough the degree of the Maillard reaction. As depicted in Figure 2b, with the increase of frozen storage time, the A_420_ of all meatballs significantly increased (*p* < 0.05). The A_420_ of samples with GLP and TP significantly reduced compared with control samples after freezing for 1 month (*p* < 0.05), and there was no significant difference between samples with GLP and samples with TP (*p* > 0.05). The increase in A_420_ was assigned to the accumulation of melanoidin produced by the Maillard reaction that kept happening during frozen storage [7]. The decrease in A_420_ was due to GLP effectively protecting the protein structures and avoiding the exposure of free amino acids, thus effectively inhibiting the Maillard reaction [36]. Zhu et al. [8] also concluded that catechin inhibited the advanced stage of the Maillard reaction.

### 3.3. Advanced Glycation end Products Formation

#### 3.3.1. Glyoxal (GO)

**Figure 3:**
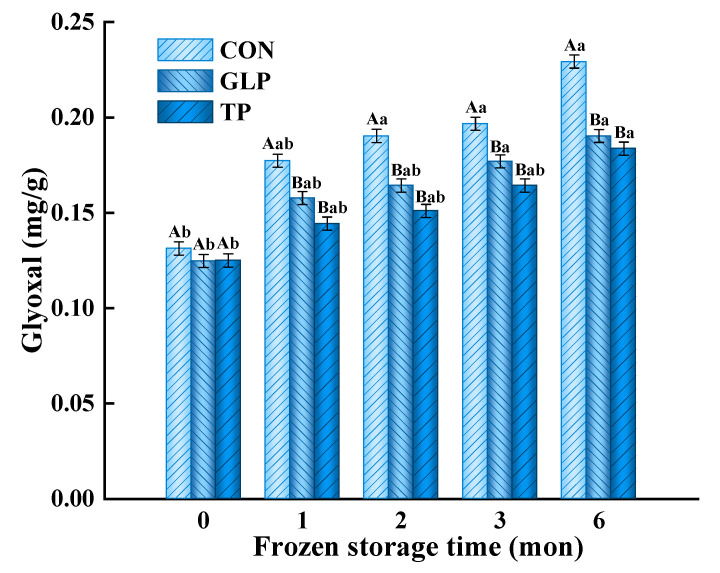
Effect of guava leaf polyphenols on the glyoxal content of frozen chicken meatballs. GLP, guava leaf polyphenols; TP, tea polyphenol; CON, control. The means at the same frozen storage with different uppercase letters (A,B) differ significantly (*p* < 0.05); the means at the same polyphenols processing with different lowercase letters (a,b) differ significantly (*p* < 0.05). The results are means ± SE (*n* = 3).

GO, a kind of α-dicarbonyl compound, is a crucial precursor substance during the formation of AGEs [40], which is produced primarily through cleavage of the Schiff base, glucose oxidation, and lipid oxidation [41]. As shown in Figure 3, the GO content of control samples increased by 42.74% when increasing the frozen time from 0 months to 6 months, while the GO content of samples treated with GLP and TP decreased by 8.27% and 10.78% compared with control samples after freezing for 6 months. The increase in GO content was mainly due to the cleavage of the Schiff base produced by the Maillard reaction, lipid oxidation, and glucose oxidation in meatballs during frozen storage [42,43,44]. The decrease in GO content was attributed to GLP and TP directly capturing GO and combining with it to generate additional products, resulting in a decrease in GO content [45]. Additionally, polyphenols in GLP inhibited α-amylase activity and decreased the content of glucose; accordingly, the content of GO was indirectly reduced [46]. Zhou et al. [47] also showed that apple polyphenols could efficiently trap reactive dicarbonyl compounds.

#### 3.3.2. N^ε^-Carboxymethyl-Lysine (CML)

**Figure 4:**
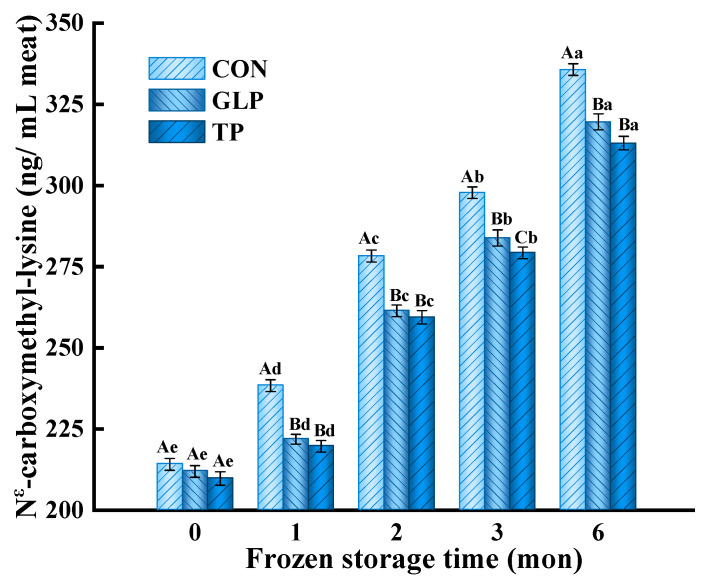
Effect of guava leaf polyphenols on the N^ε^-carboxymethyl-lysine content of frozen chicken meatballs. GLP, guava leaf polyphenols; TP, tea polyphenol; CON, control. The means at the same frozen storage with different uppercase letters (A–C) differ significantly (*p* < 0.05); the means at the same polyphenol processing with different lowercase letters (a–e) differ significantly (*p* < 0.05). The results are means ± SE (*n* = 3).

CML, a noncross-linked non-fluorescent product, is a kind of typical AGE [45]. As shown in Figure 4, with the increase of frozen storage time, the CML content of all meatballs was remarkably augmented (*p* < 0.05). After freezing for 6 months, the CML content of control samples significantly increased from 214.13 ng/mL of meat to 335.72 ng/mL of meat. Compared with the control, the CML content of samples with GLP and TP reduced by 4.80% and 6.75% after freezing for 6 months, respectively. The increase in CML content was mainly ascribed to the aggravated Maillard reaction and oxidation forming more GO during frozen storage, and the GO reacting with lysine to produce more CML; meanwhile, the aggravated Maillard reaction also generated more Schiff base that produced rearranged products by Amadori, and then the rearranged products were decomposed to form more CML [41]. The decrease in CML content was attributed to the GLP scavenging free radicals and protecting the protein structure, thus inhibiting lipid oxidation and Maillard reaction to form CML. Previous workers had also observed a decreased CML after polyphenols from grape by-products were added into muffins [11].

#### 3.3.3. Pentosidine

**Figure 5:**
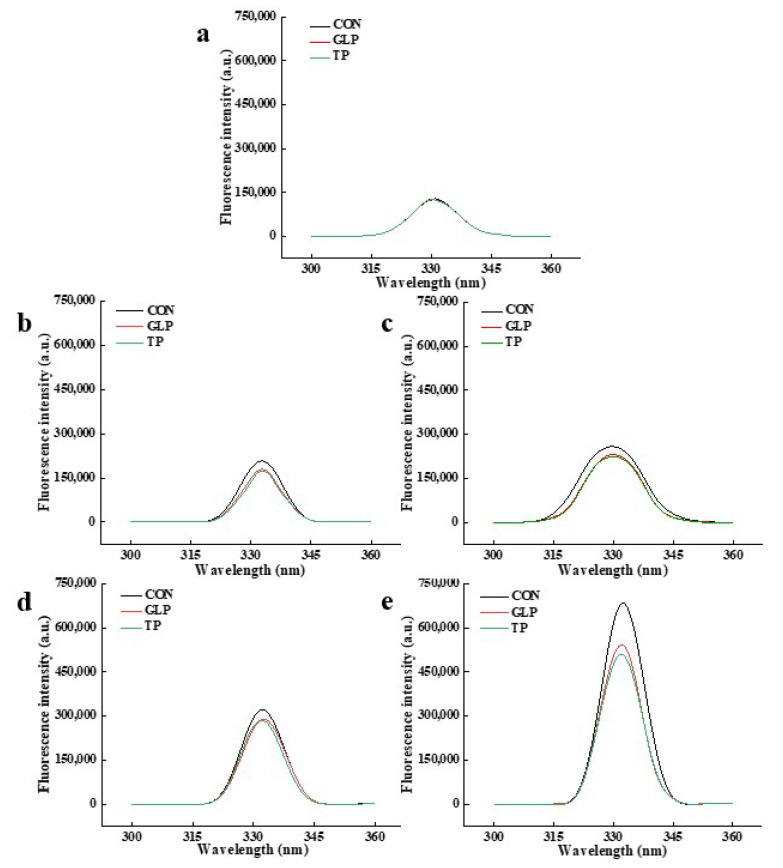
Effect of guava leaf polyphenols on the pentosidine content of frozen chicken meatballs. (**a**–**e**) indicate chicken meatballs are frozen for 0, 1, 2, 3, and 6 mon, respectively. GLP, guava leaf polyphenols; TP, tea polyphenol; CON, control.

Pentosidine, a cross-linked fluorescent product, is a kind of representative AGE which is generated by pentose reacting with the lysine and arginine [48,49]. The pentosidine content is evaluated by fluorescence intensity, and the higher the fluorescence intensity, the higher the pentosidine content. As shown in Figure 5 and Table 2, the pentosidine content of all chicken meatballs increased when increasing the frozen storage time. Compared with control samples, the pentosidine content of samples with GLP and TP decreased at the same frozen storage time. The increase in pentosidine content is owing to the hydrolysis of proteins and the continuous Maillard reaction [49]. The decrease in pentosidine content was ascribed to the ability of protecting the protein structure of GLP and TP, which inhibited the decomposition of proteins. Peng et al. [50] also obtained a similar result that cinnamon bark extracts reduced the production of pentosidine.

#### 3.3.4. Fluorescent AGEs

**Figure 6:**
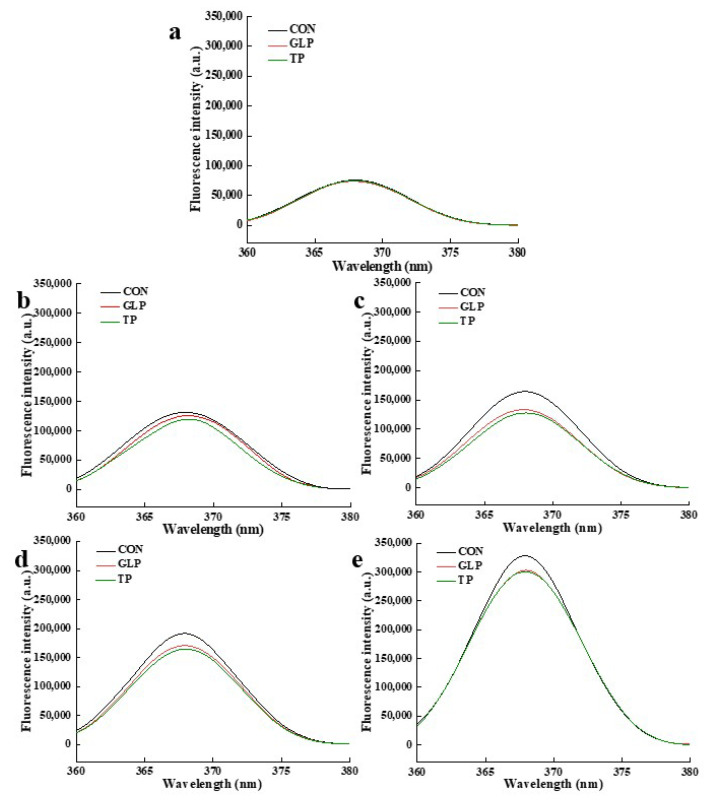
Effect of guava leaf polyphenols on the fluorescent AGEs content of frozen chicken meatballs. (**a**–**e**) indicate chicken meatballs are frozen for 0, 1, 2, 3, and 6 mon, respectively. GLP, guava leaf polyphenols; TP, tea polyphenol; CON, control.

Most of the AGEs are fluorescent products whose content is reflected by fluorescence intensity [51]. The higher the fluorescence intensity, the higher the fluorescent AGE content. As shown in Figure 6 and Table 2, with the frozen storage time increasing, the fluorescent AGE content of all chicken meatballs increased. Compared with control samples, the fluorescent AGE content of samples with GLP and TP decreased at the same frozen storage time. The increase in fluorescent AGE content was associated with the facts that (1) the Maillard reaction generated Amadori rearrangement products which were then were oxidized and cracked to form AGEs; (2) the dicarbonyl compound produced by lipid oxidation, glucose oxidation, and cleavage of the Schiff base crosslinked with proteins to generate AGEs [52,53]. The decrease in fluorescent AGEs content was related to that: (1) polyphenols directly inhibited Maillard reaction due to its abilities of protecting protein structure; (2) polyphenols reduced the production of dicarbonyl compounds; on the one hand, polyphenols directly captured dicarbonyl compounds. Zhang et al. [10] also documented the results that Chinese bayberry phenolics had excellent inhibitory effect on fluorescent AGEs.

### 3.4. Analysis of Correlation between Oxidation, Maillard Reaction, and AGEs Formation

**Figure 7:**
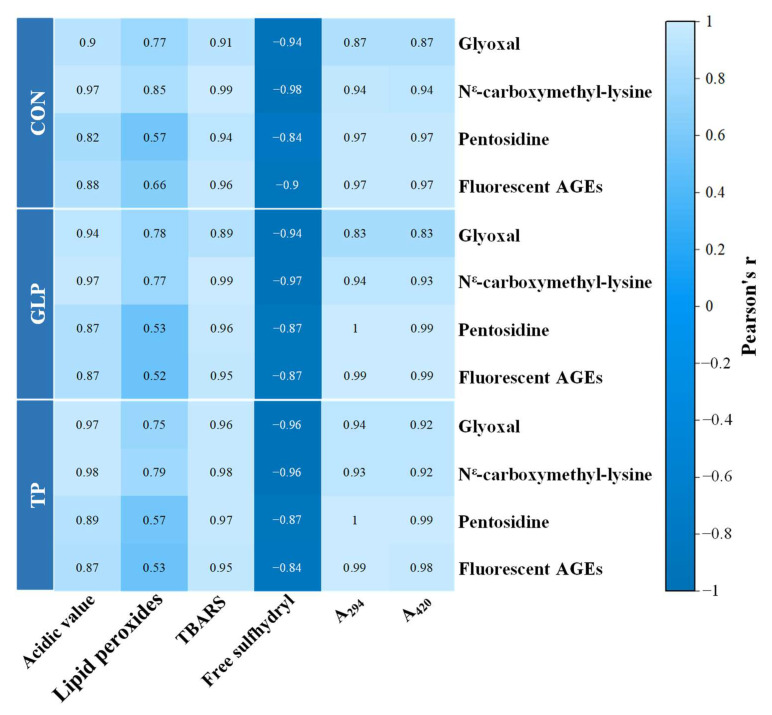
Correlation analysis of lipid oxidation, protein oxidation, and Maillard reaction with advanced glycation end product formation of frozen chicken meatballs. GLP, guava leaf polyphenols; TP, tea polyphenol; CON, control.

The correlation of lipid oxidation, protein oxidation, and Maillard reaction with advanced glycation end products formation can be analyzed by the color of grid and Pearson’s r value [54,55]. As shown in Figure 7, the heavier the blue color of the grid, the closer the Pearson’s r value was to −1, indicating a stronger negative correlation; while the lighter the blue color of the grid, the closer the Pearson’s r value was to 1, indicating a stronger positive correlation. For all chicken meatballs, there was a positive correlation: (1) between GO, CML, pentosidine, fluorescent AGEs and AV, TBARS, A_294_, and A_420_; (2) between GO, CML, and POV; while there was a negative correlation between GO, CML, pentosidine, fluorescent AGEs, and free sulfhydryl groups; and there was no correlation between pentosidine and POV.

The correlation analysis indicated that lipid oxidation, protein oxidation, Maillard reaction, and the formation of AGEs had excellent correlations. Lipid oxidation produced a variety of oxidation products, among which the dicarbonyl compound directly combined with amino acids to produce AGEs [7]. In addition, free radicals generated by lipid oxidation attacked amino acids, forming active carbonyl products (e.g., acetaldehyde and α-ketoic acid), thus promoting the Maillard reaction to produce AGEs [4,56]. Amino acid residues (e.g., lysine, etc.) produced protein oxidation directly combined with dicarbonyl compounds from Schiff base cleavage, lipid oxidation, and glucose oxidation to form AGEs [57]. The amino group exposed by protein oxidation could directly generate AGEs through the Maillard reaction, reacting with reducing sugar [4]. Thus, combined with all results, the GLP might reduce AGE formation by inhibiting lipid oxidation, protein oxidation, and the Maillard reaction. Wu et al. [4] also observed that lipid oxidation, protein oxidation, and the Maillard reaction promoted the formation of AGEs.

### 3.5. Possible Mechanism Analysis for the Inhibitory Effect of the GLP on the AGEs

**Figure 8:**
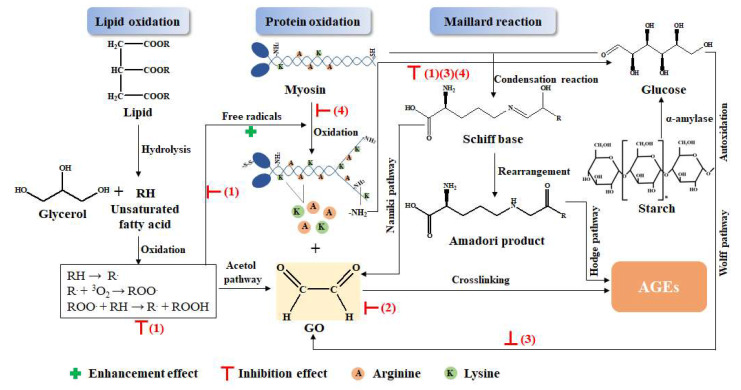
Possible mechanism for the inhibitory effect of the GLP on the AGEs.

The possible mechanism for the inhibitory effect of the GLP on the AGEs included scavenging free radicals, capturing dicarbonyl compounds, reducing the formation of glucose, and forming polyphenol–protein compounds. The graphical illustration is shown in Figure 8, where the protein is represented by myosin, which is the most abundant protein in myofibrillar proteins.

(1) GLP could scavenge free radicals produced by lipid oxidation, which stopped the chain reaction of lipid oxidation, thus preventing the formation of dicarbonyl compounds, the precursor of AGEs, and inhibiting the formation of AGEs [31]. Furthermore, protein oxidation was also prevented by scavenging free radicals; therefore, exposure of the amino group and free amino acid formation were inhibited, which reduced the substrate of the Maillard reaction and the precursor of AGEs [4].
(2) GLP directly captured dicarbonyl compounds that were the precursors of AGEs to inhibit the formation of AGEs. The benzene ring and phenolic hydroxyl group in GLP electronically conjugated to produce phenoxy anions, which could combine with dicarbonyl compounds, thus reducing the precursor of AGEs [52,53].
(3) GLP reduced the production of glucose that was the substrate of the Maillard reaction. Polyphenol in guava leaves inhibited the activity of α-amylase, which reduced the decomposition of polysaccharides into glucose, thereby reducing the substrate for the Maillard reaction [46]. In addition, GLP also reduced the content of the dicarbonyl compounds generated by glucose oxidation [45].
(4) Polyphenols in GLP combined with protein to form polyphenol–protein compounds, which reduced the Maillard reaction between proteins and sugars, resulting in decreased AGEs [11].

## 4. Conclusions

In conclusion, the effect of the GLP on the lipid oxidation, protein oxidation, Maillard reaction, and AGE formation of frozen chicken meatballs was studied. After adding GLP to chicken meatballs, the lipid oxidation, protein oxidation, degree of the Maillard reaction, and formation of AGEs were inhibited. Correlation analysis indicated that GLP inhibited AGE formation by inhibiting lipid oxidation, protein oxidation, and the Maillard reaction. The possible inhibitory mechanism of GLP on the AGEs included scavenging free radicals, capturing dicarbonyl compounds, forming polyphenol–protein compounds, and reducing the formation of glucose. This work demonstrated that plant polyphenols could be used as a natural source additive to inhibit the production of AGEs in food and provide a theoretical basis for improving the safety of industrial processed food.

## Figures and Tables

**Table 1:** The formulations of chicken meatballs.

Ingredients	Chicken Meatballs Groups		
	CON	GLP	TP
Chicken (g)	100	100	100
Pork fat (g)	20	20	20
Salt (g)	2	2	2
Potato starch (g)	6	6	6
Composite phosphate (g)	0.4	0.4	0.4
Polyphenols powder (mg)	-	9	3
Water (mL)	20	20	20

Note: “-” indicated that there was no polyphenols powder in CON.

**Table 2:** Assignments of the maximum peak of the pentosidine, and fluorescent AGEs.

Frozen Storage Time	Samples	Pentosidine		Fluorescent AGEs	
		Wavelength (nm)	Fluorescence Intensity (a.u.)	Wavelength (nm)	Fluorescence Intensity (a.u.)
0 months	CON	331	128,715	368	75,886
	GLP	331	124,415	368	73,804
	TP	330	125,327	368	75,029
1 months	CON	333	207,354	368	131,320
	GLP	333	179,418	368	125,920
	TP	333	174,050	368	119,465
2 months	CON	330	258,223	368	164,285
	GLP	330	230,923	368	133,297
	TP	330	224,132	368	127,638
3 months	CON	332	320,950	368	191,465
	GLP	333	288,865	368	170,800
	TP	332	285,138	368	164,615
6 months	CON	332	680,509	368	328,147
	GLP	332	541,299	368	302,961
	TP	332	509,650	368	300,140

## Data Availability

The data presented in this study are available in the article.
